# Human papillomavirus prevalence and type-distribution in women with cervical lesions: a cross-sectional study in Sri Lanka

**DOI:** 10.1186/1471-2407-14-116

**Published:** 2014-02-21

**Authors:** Kanishka Karunaratne, Himali Ihalagama, Saman Rohitha, Anco Molijn, Kusuma Gopala, Johannes E Schmidt, Jing Chen, Sanjoy Datta, Shailesh Mehta

**Affiliations:** 1 National Cancer Institute, Maharagama, Sri Lanka; 2 DDL Diagnostic Laboratory, Rijswijk, The Netherlands; 3 GlaxoSmithKline Pharmaceuticals Ltd, Bangalore, India; 4 GlaxoSmithKline Vaccines, Wavre, Belgium; 5 GlaxoSmithKline Vaccines, Pte Ltd, Singapore, Singapore; 6 GlaxoSmithKline Pharmaceuticals Ltd., Mumbai, India

**Keywords:** Cervical cancer, Human papillomavirus, Prevalence, Sri Lanka, Type-distribution

## Abstract

**Background:**

Cervical cancer ranks second among all cancers reported in Sri Lankan women. This study assessed the prevalence and type-distribution of human papillomavirus (HPV) among Sri Lankan women with invasive cervical cancer (ICC) and pre-cancerous lesions.

**Methods:**

114 women aged 21 years and above, hospitalized in the National Cancer Institute, Sri Lanka with a diagnosis of ICC or cervical intraepithelial neoplasia (CIN) 2/3 were prospectively enrolled between October 2009 and September 2010 (110430/NCT01221987). The cervical biopsy or excision specimens collected during routine clinical procedures were subjected to histopathological review. DNA was extracted from samples with a confirmed histological diagnosis and was amplified using polymerase chain reaction and HPV DNA was detected using Enzyme Immuno Assay. HPV positive samples were typed using reverse hybridization Line Probe Assay.

**Results:**

Of the cervical samples collected, 93.0% (106/114) had a histologically confirmed diagnosis of either ICC (98/106) or CIN 2/3 (8/106). Among all ICC cases, squamous cell carcinoma was diagnosed in the majority of women (81.6% [80/98]). HPV prevalence among ICC cases was 84.7% (83/98). The HPV types most commonly detected in ICC cases with single HPV infection (98.8% [82/83]) were HPV-16 (67.3%) and HPV-18 (9.2%). Infection with multiple HPV types was recorded in a single case (co-infection of HPV-16 and HPV-59).

**Conclusions:**

HPV was prevalent in most women with ICC in Sri Lanka; HPV-16 and HPV-18 were the predominantly detected HPV types. An effective prophylactic vaccine against the most prevalent HPV types may help to reduce the burden of ICC disease.

## Background

Worldwide disease burden data indicate that invasive cervical cancer (ICC) is the third most common type of cancer and the fourth most common cause of death due to cancer in women [1], with an estimated 530,000 new cases and nearly 275,000 deaths occurring each year [1]. According to the World Health Organization (WHO) estimates, ICC ranks as the second most common cancer among women causing approximately 1,395 new cases and nearly 814 deaths annually in Sri Lanka [2].

It is a well-established fact that persistent infection with oncogenic (or high-risk) human papillomavirus (HPV) is an important contributor for the development of ICC [3-5]. HPV can be detected in the vast majority of ICC specimens and corresponds to the highest attributable fraction as a causative agent for any major human cancer worldwide [4]. The overall prevalence of HPV in women with ICC has been reported to be as high as 99.7% around the world [4] and nearly 90% in Asia [5]. HPV-16 and HPV-18 are the most frequently reported HPV types, causing approximately 70% of ICC cases worldwide [1,3,6].

Cytological screening of the cervix using the Pap smear test and the early detection of HPV play an important role in the secondary prevention of ICC, thereby reducing HPV-associated mortality [3]. However, due to a lack of effective screening programs in lower and middle-income countries, including Sri Lanka, detection of cervical abnormalities is often difficult and leads to higher mortality rates, due to ICC, in these settings [7]. Nevertheless, recent molecular biological techniques such as HPV-DNA testing, have been found to be effective HPV screening methods and may facilitate early detection of ICC in developing regions [8].

Prophylactic vaccination represents a potential primary prevention measure against ICC [1,3]. Two prophylactic vaccines containing virus-like particles that offer protection against cervical pre-cancers and cancers are available: a bivalent vaccine (*Cervarix*™ [GlaxoSmithKline, Belgium]) containing virus-like particles for HPV-16 and -18 and a quadrivalent vaccine (*Gardasil*™ [Merck and Co., Inc, Whitehouse Station, New Jersey, USA]) containing virus-like particles for HPV-6, -11, -16 and -18 [7]. A recent systematic review and meta-analysis indicated that these vaccines are safe, well tolerated and efficacious against the vaccine-HPV types that caused persistent infection and cervical disease in young women [9]. Although both these vaccines have been licensed in Sri Lanka and are available in the private sector since 2009, they have not been included in the national immunization program [2].

Recent data on the prevalence of HPV infection and its type-distribution are limited in Sri Lanka and therefore this study was undertaken with the primary objective of assessing the prevalence of HPV-16, HPV-18 and other oncogenic HPV types among Sri Lankan women with a diagnosis of ICC and cervical intraepithelial neoplasia 2/3 (CIN 2/3). Such data is critical for assessing the potential impact of prophylactic HPV vaccines in Sri Lanka.

## Methods

### Study design and population

This cross-sectional, descriptive, observational study was conducted between October 2009 and September 2010 (110430/NCT01221987). Women aged 21 years and above who were hospitalized in the National Cancer Institute, Sri Lanka, with a confirmed histological diagnosis of ICC, squamous cell carcinoma (SCC), adenosquamous carcinoma (ASC), glandular lesions (including adenocarcinoma [ADC] and adenocarcinoma *in-situ* [AIS]), and CIN 2/3 (including carcinoma *in situ*) were prospectively enrolled. Women were excluded from the study if they had received previous vaccination against HPV, prior chemotherapy or radiotherapy for ICC, or if they had a history of recurrent episodes of ICC or CIN 2/3.

At the time of enrollment, the prevalence of HPV-16 and -18 were assumed to be 50% and 16%, respectively in ICC cases and 30% and 7%, respectively in CIN2/3 cases [10]. Assuming 10% of non-evaluable cases, an enrollment of 200 women (100 ICC and 100 CIN 2/3 cases) was planned.

This study was approved by a national Independent Ethics Committee in Sri Lanka and all the study procedures were conducted according to the principles of Good Clinical Practice, the Declaration of Helsinki version 1996 and local regulations. Written informed consent was collected from all women prior to the study conduct.

### Laboratory procedures

Cervical biopsy or excision specimens obtained during routine clinical diagnostic/operational procedures were fixed in 10% formalin solution and embedded in paraffin as tissue blocks. The review of excision specimen and verification of initial histopathological diagnosis was performed by the site pathologist and classified as ICC or CIN 2/3.

Sectioning of tissue blocks was undertaken by sandwich technique, whereby tissue sections for polymerase chain reaction (PCR) were flanked by tissue sections for histopathological review at DDL Diagnostic Laboratory, the Netherlands. A review and final diagnosis was performed by the pathologist at DDL Diagnostic Laboratory to confirm the diagnosis made by the site pathologist. If there was a disagreement between the two diagnoses, a third opinion was sought from a pathologist at DDL Diagnostic Laboratory. The final diagnosis was made by simple majority and the samples were confirmed to be ICC or CIN 2/3.

Following histopathological review and the confirmation of ICC or CIN2/3, DNA extraction was performed using proteinase K digestion [11]. Amplification of the extracted DNA was undertaken using PCR-SPF_10_ (version 1) method and HPV DNA was detected using DNA Enzyme Immuno Assay. HPV-positive specimens were typed using reverse hybridization Line Probe Assay (LiPA) using the 25 type-specific hybridization probes [11,12]. This method aided in the detection of 14 oncogenic HPV types (HPV-16, -18, -31, -33, -35, -39, -45, -51, -52, -56, -58, -59, -66 and -68) and 11 non-oncogenic HPV types (HPV-6, -11, -34, -40, -42, -43, -44, -53, -54, -70 and -74). All laboratory assays were performed at DDL Diagnostic Laboratory, the Netherlands.

### Statistical analyses

The histologically confirmed cohort included all women with confirmed diagnoses of ICC or CIN 2/3.

The percentage of women with ICC and CIN2/3 and positive for HPV-16, HPV-18 or other oncogenic HPV types was tabulated with 95% confidence interval (CI). The proportion of women positive for either HPV-16 or HPV-18 and having a co-infection with other oncogenic types and the percentage of women positive for HPV-16 and HPV-18 by histological diagnosis were also tabulated. All analyses were performed using statistical analysis system (SAS) version 9.2.

## Results

A total of 114 women were enrolled in this study, of whom 106 (93.0%) were included in the histologically confirmed cohort. A definite pathological diagnosis of ICC or CIN 2/3 was not confirmed in eight women due to insufficient ICC/CIN samples left in cervical biopsy specimen collected, thus the original cervical specimen was missing when the tissue blocks were prepared. The mean age of all enrolled women was 52.6 years (standard deviation 9.62 years); all women were of Sri Lankan origin.

### Histological diagnosis

In the histologically confirmed cohort, 92.5% (98/106) of women were diagnosed with ICC and 7.5% (8/106) were diagnosed with CIN 2/3. In women with ICC, SCC and ADC accounted for 81.6% (80/98) and 12.2% (12/98) of cases, respectively; six women were diagnosed with cervical cancer of other histological types. The histological diagnoses and the HPV infection status of women in the histologically confirmed cohort are detailed in Table 1.

**Table 1:** Histological diagnoses and HPV status of women (Histologically confirmed cohort [N = 106])

**Histological diagnosis**	**HPV status**	**n**	**Percentage**	**95% CI (LL–UL)**
All ICC n' = 98	HPV +	83	84.7	(76.0–91.2)
	Single infection	82	98.8	(93.5–100.0)
	Multiple infection	1	1.2	(0.0–6.5)
SCC n’ = 80	HPV +	72	90.0	(81.2–95.6)
	Single infection	71	98.6	(92.5–100.0)
	Multiple infection	1	1.4	(0.0–7.5)
ADC n’ = 12	HPV +	6	50.0	(21.1–78.9)
	Single infection	6	100.0	(54.1–100.0)
ASC n’ = 1	HPV -	1	100.0	(2.5–100.0)
UDC n’ = 1	HPV +	1	100.0	(2.5–100.0)
	Single infection	1	100.0	(2.5–100.0)
ON n’ = 1	HPV +	1	100.0	(2.5–100.0)
	Single infection	1	100.0	(2.5–100.0)
MIC n’ = 2	HPV +	2	100.0	(15.8–100.0)
	Single infection	2	100.0	(15.8–100.0)
AIS n’ = 1	HPV +	1	100.0	(2.5–100.0)
	Single infection	1	100.0	(2.5–100.0)
CIN 2/3 n’ = 8	HPV +	8	100.0	(63.1–100.0)
	Single infection	8	100.0	(63.1–100.0)

N: Total number of women in the histologically confirmed cohort. n: Number of women in each category. n’: Number of women by histological diagnosis. HPV+: Prevalence of HPV infection. HPV-: HPV infection not present. 95% CI: 95% confidence intervals. LL: Lower limit. UL: Upper limit. ICC, invasive cervical cancer; SCC, squamous cell carcinoma; ADC, adenocarcinoma; ASC, adenosquamous carcinoma; UDC, undifferentiated carcinoma; ON, other invasive neoplasm; AIS, adenocarcinoma *in situ*; MIC, microinvasive carcinoma; CIN, cervical intraepithelial neoplasia.

### HPV prevalence and type distribution

The overall prevalence of HPV infection in women with ICC was 84.7% ([95% CI: 76.0–91.2]; 83/98), of which 98.8% ([95% CI: 93.5–100.0]; 82/83) were infected with a single HPV type and 1.2% ([95% CI: 0.0–6.5]; 1/83) had multiple HPV type infection (Table 1).

Among women with SCC and ADC, HPV prevalence was 90.0% ([95% CI: 81.2–95.6]; 72/80) and 50.0% ([95% CI: 21.1–78.9]; 6/12), respectively (Table 1).

**Figure 1:**
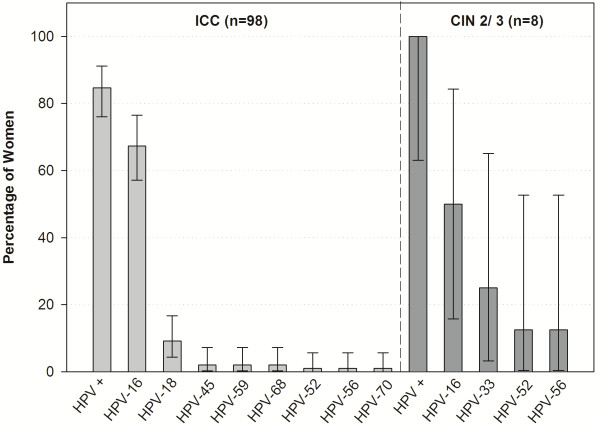
**HPV prevalence and type distribution among adult women with cervical lesions (Histologically confirmed cohort [N = 106]).** Note: The error bars indicate the 95% confidence intervals. HPV-45, -59 and -68 accounted for 2% women each and HPV-52, -56 and -70 accounted for 1% women each.

The HPV type distribution among women with ICC, by histological diagnosis, is illustrated in Figure 1. HPV-16 was detected in 67.3% women ([95% CI: 57.1–76.5]; 66/98) and HPV-18 in 9.2% ([95% CI: 4.3–16.7]; 9/98). Other oncogenic HPV types included HPV-45, HPV-59 and HPV-68 each was detected in two women (2.0% [95% CI: 0.2–7.2]), and HPV-52, HPV-56 and HPV-70 each detected in one women (1.0% [95% CI: 0.0–5.6]) (Figure 1). Co-infection of HPV-16 and HPV-59 was observed in a single case (1.5% [95% CI: 0.0–8.2]) of ICC. All eight CIN 2/3 cases were HPV positive (100.0% [95% CI: 63.1–100.0]), with HPV-16 being the most predominant type detected (50.0% [95% CI: 15.7–84.3]) followed by HPV-33 (25.0% [95% CI: 3.2–65.1]), HPV-52 and HPV-56 (12.5% [95% CI: 0.3–52.7], respectively) (Figure 1).

Non-oncogenic HPV types were not detected in any woman in the histologically confirmed cohort.

## Discussion

This descriptive observational study provides the most recent data on the prevalence and type distribution of HPV in Sri Lankan women aged 21 years or more with a diagnosis of ICC. The overall prevalence of HPV in women with histologically confirmed ICC in our study (84.7%) was lower than the 93% rate previously reported by Samarawickrema et al. [13] in a retrospective study using archival cervical tissue samples to assess HPV type distribution in a similar Sri Lankan population. Samarawickrema et al. employed reverse hybridization technique using INNO-LiPA HPV Genotyping Extra Kit (Innogenetics, Gent, Belgium) for HPV DNA detection and typing [13], which is a modified version of the original SPF_10_- DEIA/LiPA_25_ PCR system (SPF_10_-LiPA_25_ version 1 [licensed Innogenetics technology, manufactured by Labo Biomedical Products, Rijswijk, The Netherlands]) [14] which we used. In addition to using similar laboratory procedures, Samarawickerma et al. performed HPV typing on all ICC cases [13], which is similar to the present study. However, Samarawickerma study [13] used archival cervical biopsy specimens to assess the prevalence and type-distribution of HPV which is in contrast to the design of the present study. Although this could partly explain the difference in overall HPV prevalence rates between the two studies, the exact reason is uncertain.

In the present study, the HPV prevalence in women with SCC was 90.0%, which is in line with a meta-analysis of published literature in Asian women with SCC where the adjusted overall HPV prevalence was 86.1% [5].

HPV-16 (67.3%) and HPV-18 (9.2%) were the most prevalent HPV types in ICC cases. This corresponds to worldwide estimates of approximately 70% of ICC cases which are caused by these HPV types [1,3]. The prevalence of HPV-16 reported in this study was lower when compared to other studies in Sri Lankan women with invasive SCC (74%–77%) [13,15], but higher than that reported from a worldwide surveillance study by Sanjose et al. (61%) [16]. On the other hand, the prevalence of HPV-18 in this study was lower when compared to that reported by de Silva et al. (20%) [15] and Sanjose et al. (10%) [16] but higher than that reported in a study by Samarawickrema et al. (6.5%) [13]. This highlights the need for a vaccine which offers broader protection, irrespective of type, as non-HPV-16/-18 lesions may play a substantial role in the local disease burden [17].

Although a meta-analysis of global studies found that infection with multiple HPV types is common [18], in the current study, only one woman with ICC was infected with multiple HPV types. A similar observation was made in a previously conducted study in Sri Lanka where only one case with multiple HPV type infection was reported amongst 108 women diagnosed with invasive SCC [13]. However, the exact reason for the observed fewer multiple infections are unknown.

The strengths of this study include the confirmation of histopathological diagnosis of ICC and/or CIN 2/3 cases by a centralized pathology laboratory and using the highly sensitive SPF_10_-LiPA assay for detecting HPV DNA, which provides scientific validity and ensures comparability of the results with studies conducted worldwide using similar methodologies. Since the cervical specimens were prospectively collected, they were more recent and might have resulted in higher HPV DNA detection rates as compared to studies which use archival specimens [13,16].

However, the results have to be interpreted with caution due to several limitations. Firstly, the present study evaluated only HPV positive ICC cases and did not investigate the extent to which HPV negative cases might in fact be false negatives. Among ICC cases in this study, 15.3% (15/98) tested negative for HPV DNA. Failure to detect HPV DNA in ICC cases, despite persistent HPV infection being postulated to be a "necessary cause" of ICC, may be due to inadequate sampling or disruption of the PCR target sequence due to viral integration [4]. Such an observation was made by Walboomers and colleagues when they re-assessed HPV negative ICC specimens from the International Biological Study on Cervical Cancer study [19] and concluded that at least 40 of the 66 HPV-negative ICC specimens tested using MY09/11 PCR assay were indeed false negatives [4]. Secondly, the number of CIN 2/3 cases enrolled was lower than planned (100 CIN 2/3 cases) because the recruiting centers were tertiary hospitals which mainly treated invasive cancers. In Sri Lanka, structured and organized cervical screening programs are lacking and cervical smears are only collected opportunistically [20]. As a consequence, only the most severe ICC cases are likely to be detected. Furthermore, the ineffective screening programs could result in the relatively lower detection rates of pre-cancerous cases which in turn might have led to small number of CIN 2/3 cases enrolled in this study. Therefore, because of selection bias, the selected sample size is non-representative of the population and further studies with sufficient cases will be required to better understand the prevalence and type distribution of HPV infection among precancerous lesions. Lastly, although the prevalence and distribution of HPV types might vary among histological diagnosis of ICC (SCC and ADC), the primary objective of the study was to assess the prevalence and distribution of HPV types among all ICC cases only. In addition, the number of ADC cases in the study was low (n = 12) thus no conclusion on HPV prevalence in ADC could be drawn.

WHO recommends that HPV vaccination be included in the national immunization programs of countries where ICC and/or other HPV-related diseases constitute a public health priority [2]. Recently conducted clinical trials of the bivalent and quadrivalent vaccines indicated that both vaccines had high efficacy against CIN in women who were not previously infected with HPV [21,22]. Indeed, in Sri Lanka, the nationwide introduction of effective prophylactic vaccines, which provide protection against the prevalent HPV types, might help to effectively reduce the long-term HPV-associated disease burden of ICC. Sri Lanka has a considerably lower maternal mortality ratio compared to other countries in South Asia (1 in 430 as compared to 1 in 43, respectively) [23], which is the direct result of improved education and cost-free health care, advances in knowledge and infrastructure of healthcare facilities, improvements in nutrition, sanitation and immunization practices [24]. The implementation of a comprehensive strategy, combining HPV vaccination together with effective cervical screening programs might substantially help to enhance women’s health in Sri Lanka.

## Conclusions

HPV infection was prevalent in the majority of Sri Lankan women with ICC. HPV-16 and HPV-18 were the predominant HPV types in this population. An effective prophylactic vaccine, which offers protection against oncogenic HPV types, may therefore help to reduce the disease burden of ICC in a comprehensive cervical cancer prevention program.

### Trademark statement

*Cervarix* is a registered trademark of the GlaxoSmithKline group of companies.

*Gardasil* is a trademark of Merck & Co., Inc.

## Competing interests

Authors JC, SM, KG, JS and SD declare that they are employed by the GlaxoSmithKline group of companies and JC, JS, SM and SD hold stocks. AM declares to have received payment for consultation, laboratory based sample testing and data analysis and study-related travel from the GlaxoSmithKline group of companies. Authors KK, SR and HI declare to have no conflicts of interest to declare.

## Authors’ contributions

KK was the Principal Investigator of the study involved in conception of the study, collection of cervical biopsy specimens, administering informed consent and led the team of co-investigators in conducting the study. HI was the co-investigator involved in data collection and was involved in supervision of subjects selected and recruited into the study. SR was the co-investigator who was involved in collection of cervical biopsy specimens, administering informed consent and collection and assembly of data. AM was the scientist at the HPV testing laboratory who was involved in study set-up, supervised the pathological diagnosis and lab testing done at DDL diagnostic laboratories and interpreted the lab results from HPV pathology testing. KG was the statistician who performed data analysis and also interpreted the results of the study. JS was the epidemiologist involved in conception of the study and interpretation of study results. JC was the epidemiologist involved in conception of the study. SD was the medical monitor involved in conception of the study, collection of data, acquisition of funding, recruitment of investigators and supervision of research group. SM was the study responsible person and he was involved in conception of the study and collection of data. All authors were involved in review of the manuscript and approval of the final content prior to submission.

## Pre-publication history

The pre-publication history for this paper can be accessed here:

http://www.biomedcentral.com/1471-2407/14/116/prepub
